# Nootropic and Neuroprotective Effects of *Dichrocephala integrifolia* on Scopolamine Mouse Model of Alzheimer’s Disease

**DOI:** 10.3389/fphar.2017.00847

**Published:** 2017-11-21

**Authors:** Nadège E. Kouémou, Germain S. Taiwe, Fleur C. O. Moto, Simon Pale, Gwladys T. Ngoupaye, Jacqueline S. K. Njapdounke, Gisèle C. N. Nkantchoua, David B. Pahaye, Elisabeth Ngo Bum

**Affiliations:** ^1^Department of Zoology and Animal Physiology, Faculty of Science, University of Buea, Buea, Cameroon; ^2^Department of Biological Sciences, Faculty of Science, University of Ngaoundéré, Ngaoundéré, Cameroon; ^3^Department of Biological Sciences, Higher Teachers’ Training College, University of Yaoundé I, Yaoundé, Cameroon; ^4^Department of Animal Biology, Faculty of Science, University of Dschang, Dschang, Cameroon; ^5^Institute of Mines and Petroleum Industries of Maroua, University of Maroua, Maroua, Cameroon

**Keywords:** *Dichrocephala integrifolia*, Alzheimer’s disease, memory impairment, behavior, scopolamine, acetylcholinesterase inhibitor, oxidative stress

## Abstract

Alzheimer’s disease the most common form of dementia in the elderly is a neurodegenerative disease that affects 44 millions of people worldwide. The first treatments against Alzheimer’s disease are acetylcholinesterase inhibitors; however, these medications are associated with many side effects. *Dichrocephala integrifolia* is a traditional herb widely used by indigenous population of Cameroon to treat and prevent Alzheimer’s disease and for memory improvement. In this study, we evaluated the effect of the decoction prepared from leaves of *D. integrifolia*, on scopolamine-induced memory impairment in mice. Seven groups of six animals were used. The first two groups received distilled water for the distilled water and scopolamine groups. The four test groups received one of the four doses of the decoction of the plant (35, 87.5, 175 or 350 mg/kg p.o.) and the positive control group received tacrine (10 mg/kg), a cholinesterase inhibitor used in the treatment of Alzheimer’s disease, during 10 consecutive days. Scopolamine (1 mg/kg), a cholinergic receptor blocker, administered 30 min after treatments, was used to induce memory impairment to all groups except the distilled water group on day 10 of drug treatment. The behavioral paradigms used to evaluate the effects of the treatment were the elevated plus maze for learning and memory, Y maze for spatial short-term memory, the novel object recognition for recognition memory and Morris water maze for the evaluation of spatial long-term memory. After behavioral tests, animals were sacrificed and brains of a subset were used for the assessment of some biomarkers of oxidative stress (malondialdehyde and reduced glutathione levels) and for the evaluation of the acetylcholinesterase activity. From the remaining subset brains, histopathological analysis was performed. The results of this study showed that, *D. integrifolia* at the doses of 87.5 and 350 mg/kg significantly (*p* < 0.01) improved spatial short-term and long-term memory, by increasing the percentage of spontaneous alternation in the Y maze and reducing the escape latency in the Morris water maze. Furthermore, the results of histopathological evaluation showed that *D. integrifolia* attenuated the neuronal death in the hippocampus induced by scopolamine. The main finding of this work is that *D. integrifolia* improves learning capacities and counteracts the memory impairment induced by scopolamine. Thus, *D. integrifolia* can be a promising plant resource for the management of Alzheimer’s disease and memory loss.

## Introduction

Alzheimer’s disease (AD) the most common form of dementia in the elderly, is a neurodegenerative disease that is clinically characterized by progressive memory loss, cognitive dysfunction and reduction of learning capacities with increase age (Baulac et al., 2003; Cheng et al., 2011; Terry et al., 2011; Alzheimer’s Association, 2016). The number of persons affected by dementia worldwide is estimated at 47 million and AD represents 60–80% of this number (Alzheimer’s Association, 2016; Alzheimer’s Disease International, 2016). Neurofibrillary tangles and neuritic plaques are the two main pathological hallmarks of AD. The AD brain is also characterized by a reduction in cholinergic neurotransmission and an increase in oxidative stress (Francis et al., 1999). The evidence of oxidative stress in the brains of AD patients is the increase of the end products of lipid peroxidation, like malondialdehyde and a reduction in antioxidant enzymes: glutathione and superoxide dismutase (Christen, 2000; Padurariu et al., 2013). The reduction in the cholinergic transmission appears to be the critical element producing dementia. The first treatments against AD are acetylcholinesterase inhibitors, which enhance the cholinergic neurotransmission by increasing the availability of acetylcholine in cholinergic synapse (Giacobini, 2000). Acetylcholinesterase inhibitors have failed in the treatment of AD because of their limited efficacy and bioavailability and because they are associated which many side effects such as hepatotoxicity (Kulkarni et al., 2011). Due to the growing population and extended lifespan, dementia of the type Alzheimer have become a major health concern in the elderly in Africa (Kalaria et al., 2008; Olayinka and Mbuyi, 2014). *Dichrocephala integrifolia* is a plant of the family Asteraceae that is widely used in traditional medicine in Cameroon to treat and prevent dementia and Alzheimer disease (Ngueguim et al., 2016). In west Cameroon, *D. integrifolia* is known as “Mbag’api” and the decoction of its leaves is used to treat dementia. The indigenous populations of central region of Cameroon call it “Ngninada Elokn” and use the infusion of the whole plant to treat memory impairment. In far north Cameroon where *D. integrifolia* is called “Ganki” the decoction prepared from its leaves is used in the treatment of Alzheimer disease (Personal communication). Despite the vast empirical knowledge about the uses of *D. integrifolia* in the treatment of dementia in Cameroon, pharmacological studies to validate its use in the treatment of dementia are scarce. Thus, the aim of the present study was to evaluate the effect of the decoction of the leaves of *D. integrifolia* on scopolamine mouse model of Alzheimer’s disease.

## Materials and Methods

### Animals

The animals used in this study were Swiss mice of either sex weighing 25–30 g. The mice were bred in the animal house of the Faculty of Science of the University of Buea, under a 12 h light/dark cycle. The mice, grouped 6 per cage had food and water available *ad libitum*. The mice were acclimatized to laboratory conditions for 24 h before the beginning of experimentations. The study was conducted in accordance with the Cameroon National Committee (Ref No. FW-IRB00001954), and was authorized under a number CEI-UDo/908/01/2017/T, and in conformation with the international regulation. All efforts were taken to minimizing the number of mice used and their suffering. Behavioral procedures were performed between 9 a.m. and 4 p.m.

### Plant Material

#### Collection and Identification

*Dichrocephala integrifolia* leaves were harvested in April 2014 in the South–West Region of Cameroon. The harvesting coordinates are 4°15′30″and 9°25′48″. The botanical identification of the plant was done at the National Herbarium of Cameroon, where a voucher specimen was conserved under the reference number: 24276/SRFcam.

#### Preparation of the Decoction of *D. integrifolia*

The leaves of *D. integrifolia* were cleaned, shade-dried and ground. The decoction of *D. integrifolia* was prepared daily according to the instructions of the native doctor. Ten (10 g) of the leaves’ powder of *D. integrifolia* were introduced in 75 ml of distilled water, the mixture was then boiled for 20 min. After cooling, the mixture was filtered using Whatman N° 1 filter paper. The filtrate constituted the stock solution. In another experiment, 20 ml of the stock solution was evaporated to dryness and the dry residue obtained was 700 mg. The corresponding concentration of the stock solution was 35 mg/ml. The stock solution was diluted 2; 4; and 10 times in distilled water for less concentrated solutions. All solutions were administered to mice in a volume of 10 ml/kg body weight. The corresponding dose for the stock solution as described by the traditional healer was 350 mg/kg. The doses of the different dilutions were 175; 87.5; and 35 mg/kg.

### Drugs and Chemicals

Tacrine (9-amino-1, 2, 3, 4-tetrahydroacridine hydrochloride), scopolamine hydrobromide, trichloroacetic acid and thiobarbituric acid were purchased from Sigma chemical, St. Louis, MO (United States). Acetylthiocholine iodide, and 5, 5′-dithiobis (2-nitrobenzoic acid) (Ellman reagent) were purchased from Biochemica (China).

### Study of the Effect of *D. integrifolia* on Memory Impairment Induced by a Single Dose of Scopolamine

This test was used to assess the effect of the decoction of *D. integrifolia* administered for 10 consecutive days against memory impairment induced by a single injection of scopolamine at the dose of 1 mg/kg i.p. The behavioral tasks used to evaluate the effect of the treatment were the Y maze, the elevated plus maze and the novel object recognition task.

#### General Experimental Design

In this part of the work, mice were randomly divided into seven groups of six mice each (three males and three females) and group as follow:

(1) Group I: The distilled water group; which received only distilled water (10 ml/kg) orally;
(1) Group II: Scopolamine group; which received distilled water (10 ml/kg) orally;
(1) Groups III–VI: Tests groups; which received one of the four doses of the decoction of *D. integrifolia;* 350, 175; 87.5, or 35 mg/kg, orally
  Groups VII: Tacrine group; which received tacrine at the dose of 10 mg/kg orally.

All these groups received the corresponding treatment for 10 consecutive days. On day 10, 30 min after the various treatments, scopolamine (1 mg/kg i.p.) was injected to all groups except the distilled water group that still received distilled water. The behavioral tests were performed 30 min after the injection of scopolamine.

#### Behavioral Assessment

##### Y-maze test

Y-maze test was used to evaluate short-term memory of mice by recording spontaneous alternation in a single session on day 10. The maze used in this study was a Y-maze made of polywood with three identical arms (35 cm length × 8 cm height × 15 cm width) mounted at 120 degrees to one another in a single piece. Each arm of the Y-maze was decorated with a different letter (A, B, or C) in order to be differentiated (Ma et al., 2007). One hour after the last treatment and 30 min after scopolamine injection (except for the distilled water group), each mice, previously naive to the maze, was placed at the end of one arm and were allow to move freely through the maze during 8 min. The number of arm entries was recorded for each mouse. An arm entry was noted when a mouse entered an arm of the maze with all its paws. Specific sequences of arm transitions (ABC, BCA, or CAB but not BAB or CAC or CBC) were recorded as a spontaneous alternation that reflects short-term memory. The total number of arm entries reflects general locomotor activity. The arms of the maze were cleaned between sessions with 10% ethanol. The percentage of spontaneous alternation was defined according to the following equation:percentage of spontaneous alternation = [(Number of alternations)/(Total arm entries - 2)] × 100 (Ma et al., 2007; Hritcu et al., 2012; Beppe et al., 2014).

##### Elevated plus-maze test

Elevated Plus Maze (EPM) is an exteroceptive behavioral model used to evaluate learning and memory in rodents (Itoh et al., 1990; Sharma and Kulkarni, 1992; Kulkarni et al., 2011). The EPM was in plywood and comprised two open arms (30 cm × 5 cm) and two closed arms (30 cm × 5 cm × 15 cm) that extended from a common central platform (5 cm × 5 cm). The maze was elevated to a height of 50 cm above the floor level.

On the 1st day of the test (day 9 of drug treatment), 1 h after various treatments, each mouse was placed at the end of an open arm, facing away from the central platform for a learning trial. Transfer latency (TL) was taken as the time taken by the animal to move into any one of the covered arms with all its four limbs. The cutting time was 120 s after this time an animal that did not enter into one of the covered arms was gently push into one and the TL was assigned as 120 s. The mouse was allowed to explore the maze for another 2 min. On the 2nd day of the test (day 10 of drug treatment), during retention phase, retention transfer latency was recorded 1 h after the last treatment and 30 min after scopolamine injection (except for the distilled water group). Reduction in TL values of the 2nd day of the test in comparison to the 1st day test indicates improvement in memory (Itoh et al., 1990; Sharma and Kulkarni, 1992; Joshi and Parle, 2007; Kulkarni et al., 2011).

##### The novel object recognition test (NOR)

The NOR test was used to evaluate recognition memory of mice. This test was performed in an open field apparatus consisted of square plywood of dimensions (40 cm × 40 cm × 25 cm). The day before the test (day 8 of drug treatment), 1 h after drug treatment, each mouse individually was familiarized with the apparatus for 5 min. On the 1st day of the test (day 9 of drug treatment) 1 h after drug treatment, two identical objects were presented to each mouse for a 5 min session of exploration. An animal explores an object when it touches the object or it directs its nose at a distance less than 2 cm to the object. The 2nd day of the test (day 10 of drug treatment), 30 min after scopolamine injection (except for the distilled water group), a new object replaced one of the objects presented in the 1st day. The time spent by the animal for exploring the new object (tB) and the familiar (tA) objects was recorded during 5 min (Kulkarni et al., 2011). The discrimination index (DI) was calculated as (tB/tB+tA) (Ennaceur and Delacour, 1988; Rajendran et al., 2014).

### Study of the Effect of *D. integrifolia* against Memory Impairment Induced by Repeated Doses of Scopolamine

#### Experiment Design

To delineate the mechanism by which *D. integrifolia* exerts its protective effect, in a subsequent test, the mice were divided into seven groups of six as described above. Scopolamine (1 mg/kg i.p.) were injected to all the groups every day for 10 days 1 h after 30 min after drug administration except the distilled water which received injection of saline (10 ml/kg ip).

The behavioral task used to evaluate the effect of the treatment was Morris water maze task (MWM).

#### Behavioral Assessment: The Morris Water Maze Task

The MWM test was used to evaluate spatial long-term memory of mice. The MWM was performed as previously described by Morris in 1984 with little modifications (Morris, 1984; Parle and Singh, 2007). The MWM consisted of a brown circular pool (100 cm diameter, 50 cm height). The pool was located in a room with various visual cues (pictures, shelters, curtains, lamps, etc…). The position of the pool and that of the cues were maintained all the days of the experimentations. The pool was filled with water at the temperature of 25 ± 2°C. The MWM was virtually divided into four equal quadrants: North, South, East, and West. A platform of white color (11 cm diameter and 16 cm height) was centered in the South–East quadrant 1 cm below the water surface. The water was whitened by addition of liquid milk so that the platform was invisible at water surface. The position of the platform was kept unaltered during the training session. The 1st day of the MWM test (day 6 of drug treatment), 1 h after drug administration and 30 min after scopolamine injection, each mouse received an acclimatization session during which, the mouse was placed inside the MWM for swimming for 60 s. During the acquisition phase (days 7–9 of drug treatment), 30 min after scopolamine injection, each mouse was released into the pool, head facing the wall. The cutting time for each trial was 120 s. each mouse that did not find the platform during the time was gently guide to it and allowed there for 10 s. Each animal had four training sessions per day of 5 min interval. After each trial each mouse was taken to its cage and was allowed to dry up under a 60 watt bulb.

During each trial session, the time taken to reach the platform (escape latency) was recorded with stopwatches. In the retention phase (day 10 of drug treatment), the platform was removed from the pool. Each mouse individually was placed into the MWM. The latency time taken to reach the place of the formal platform and the time spent in the target quadrant was recorded during 120 s using stopwatches by experimenter researchers.

#### Biochemical Assays

On day 11 following the MWM test, mice were decapitated under light ether anesthesia. In each group, the brain of a sub set of animal was used for histopathological analysis and the other for the dosage of brain malondialdehyde (a marker of lipid peroxidation), reduced glutathione (the principal antioxidant enzyme of the body) and acetylcholinesterase activity (which give an idea on brain acetylcholine level).

##### Tissue preparation

After sacrifices, the brains were immediately removed, from the skull, rinsed and weighed. Each brain was divided into two cerebral hemispheres. Brain homogenate was prepared from one half with 50 mM Tris/HCl buffer for the assessment of brain malondialdehyde (MDA) and reduced glutathione levels. For the assessment of acetylcholine esterase activity, the other hemisphere of the brains was used to prepare 10% homogenate with 50 mM Tris/HCl buffer containing 1% Triton –X.

##### Estimation of protein concentration

The total protein of brains homogenate was determined by the method described by Bradford (1976). Five (5) μl of the brain homogenate was introduced in microplate wells and 250 μl of Bradford reagent was added. After agitation, the absorbance of the mixture was read using a microplate reader at 590 nm. The determination of the protein concentration was done using bovine serum albumin (BSA) as standard.

##### Determination of brain acetylcholinesterase activity

The determination of brain acetylcholinesterase activity was performs using acetylthiocholine iodide as artificial substrate based on the colorimetric method, of Ellman (Ellman et al., 1961). Briefly, 925 μL of 0.5 mM of Ellman reagent prepared in 100 mM Tris buffer (pH.8) was mixed with 50 μL of 20 mM acetylthiocholine iodide and 25 μL of supernatant. The change in absorbance was monitored using a spectrophotometer during 3 min at 30 s interval. The activity of AchE is expressed as AChE activity is expressed as micromoles of acetylthiocholine iodide hydrolyzed per milligram of protein per minute. Each assay was done in duplicate.

##### Brain reduced glutathione level

Reduced glutathione (GSH) level was estimate in the brain supernatant according to the method of Ellman (1959). Twenty (20) μl of brain homogenates were mixed with 3 ml of Ellman reagent prepared in phosphate buffer (0.1 M pH 7.2) at room temperature. After 1 h, the absorbance of the mixture was read at 412 nm. The amount of glutathione was calculated with the formula of Beer Lambert using the extinction coefficient value of 13,600/M/cm (Fotio et al., 2009). Each assay was done in triplicate.

##### Brain malondialdehyde level

The brain malondialdehyde (MDA) level was measured in the supernatant using the thiobabituric assay. One (1 ml) of brain’s supernatant was added to 0.5 ml of trichloroacetic acid (20%) and 1 ml of thiobarbituric acid (0.67%). The mixture was heated in a water bath at 100°C for 60 min. After cooling, the mixture was centrifuged at 3000 rpm for 15 min. The absorbance of the supernatant was read at 530 nm. The amount of MDA was calculated with the formula of Beer Lambert using the extinction coefficient value of 1.56 × 10^5^ M/cm. The concentration of MDA is expressed as μmol/g tissue (Nelson et al., 1994; Fotio et al., 2009). Each assay was done in triplicate.

#### Histopathological Studies

After sacrifices, the brains were fixed in 10% formol for a week. Fifty (50) μm coronally sections were made from the brain in the hippocampus region using the mouse brain Atlas with the following coordinate (Anterior/Posterior = -2.0 mm, Medial/lateral = -1.5 mm, and dorsal/ventral AP = -2.0 mm) (Paxinos and Franklin, 2001). The brains sections were collected in nine well plates. The dehydration of brain section consisted in introducing brain session in ascending concentration of ethanol and then followed by immersion in xylol and then embedding in paraffin. Paraffin sections of the brain were deparaffinized and rehydrated through washes in descending concentration series of alcohol. Brain sections were then stained using the Nissl stain. After drying overnight, the brain sections were photographed and images were captured using a digital camera attached to a light microscope.

### Statistical Analysis

Statistical analysis was done using the software Graphpad Instat for windows. The differences amongst groups were analyzed using One-Way Analysis of Variance (ANOVA). *P*-values ≤ 0.05 were considered significant. Tukey *post hoc* test were used for multiple comparisons.

## Results

### Effects of *D. integrifolia* on Spontaneous Alternation of Scopolamine Treated Mice in the Y Maze

**FIGURE 1:**
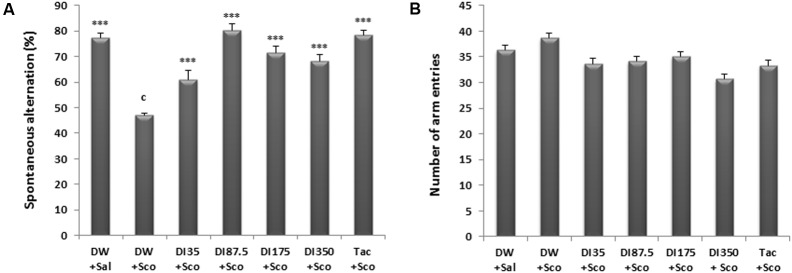
Effect of *Dichrocephala integrifolia* on scopolamine-induced memory impairments in the Y-maze test. The spontaneous alternation percentage **(A)** and the numbers of arm entries **(B)**. Each column represents mean ± SEM of six mice. Data analysis was performed using one way ANOVA followed by Tukey multi comparison test, ^∗^*P* ≤ 0.01, ^∗∗∗^*P* ≤ 0.001 vs. scopolamine treated group (DW + Sco); ^c^*P* < 0.001 vs. distilled water group. DW, distilled water; DI, *D. integrifolia*; Sco, scopolamine; Tac, tacrine.

The results of spontaneous alternation show that there was a significant difference among all the treatments groups (*P* < 0.0001). Scopolamine reduced the mice spontaneous alternation. Ten days treatment with *D. integrifolia* at all doses significantly reversed the effect of scopolamine and increased the spontaneous alternation percentage (*P* < 0.0001), when compared to scopolamine-alone treated group. Tacrine 10 mg/kg pre-administration also reversed the reduction of spontaneous alternation induced by scopolamine (**Figure 1A**). Tacrine reversed the effects of scopolamine at the percentage of 78.59% and the decoction at the dose of 87.5 mg/kg at a percentage of 80.21%.

*Dichrocephala integrifolia* did not increased or impaired the locomotion of scopolamine treated mice in the Y maze (**Figure 1B**).

### Effects of *D. integrifolia* on Transfer Latencies in an Elevated Plus Maze of Scopolamine-Treated Mice

**FIGURE 2:**
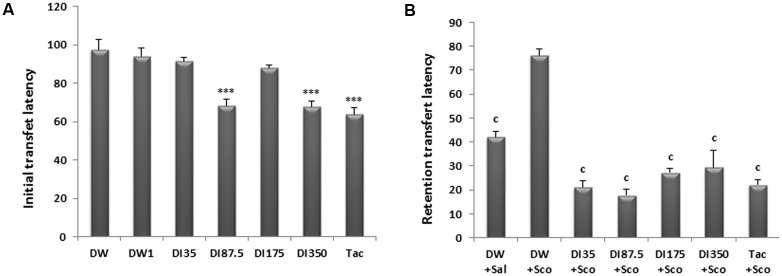
Effect of *D. integrifolia* on scopolamine-induced memory impairment on transfer latencies using elevated plus maze. Initial transfer latency (s) on day 9 **(A)** and retention transfer latency (s) on day 10 **(B)**. Each column represents mean ± SEM of six animals. The data analysis was performed using one way ANOVA followed by turkey multiple comparisons test, ^∗∗∗^*P* < 0.001; vs. distilled water treated group that will not receive scopolamine on day 10 (DW0). ^c^*P* < 0.01 vs. distilled water treated group that receive scopolamine on day 10 (DW1). DI, *D. integrifolia*; Sal, saline; Sco, scopolamine; Tac, tacrine.

As shown in **Figure 2A**, there was a significant difference among groups concerning the initial transfer latency. The decoction of *D. integrifolia* at the doses of 87.5 and 350 mg/kg decreased the initial transfer latency to 68.33 ± 3.33 s and 67.66 ± 3.04 s, respectively, against 97.5 ± 5.12 s in the distilled water group at day 9. Tacrine group had also a short initial transfer latency of 63.83 ± 3.39 s on day 9 when compared to distilled water group (*P* < 0.0001).

A single administration of scopolamine significantly increased the retention transfer latency (RTL, recorded on the second testing day) of scopolamine alone treated group when compared to distilled water group. This RTL was 42.16 ± 2.07 s in the distilled water group against 76.16 ± 2.49 s in the scopolamine alone treated group. The decoction of *D. integrifolia* at all the doses significantly (*P* < 0.0001) reduced the RTL. Tacrine also reversed the retention deficit induced by scopolamine on day 10 of treatment (**Figure 2B**).

### Effects of *D. integrifolia* on Recognition Memory of Scopolamine Treated Mice

**FIGURE 3:**
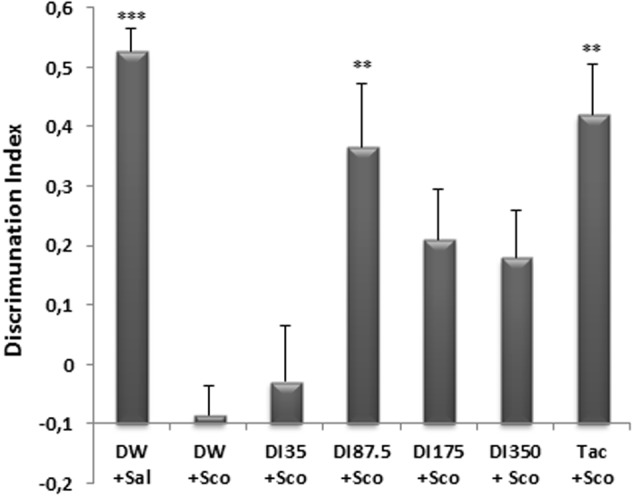
Effect of *D. integrifolia* on scopolamine-induced memory impairment on Discrimination Index in object recognition test. Each column represents mean ± SEM of six animals. The data analysis was performed using one way ANOVA followed by Turkey multiple comparisons test. ^∗∗^*P* < 0.01, ^∗∗∗^*P* < 0.001 vs. Scopolamine treated group (DW + Sco). DW, distilled water; DI, *D. integrifolia*; Sal, saline; Sco, scopolamine; Tac, tacrine.

The NOR test was used to assess recognition memory of mice after a single injection of scopolamine. The administration of scopolamine before the retention phase of the test resulted in a reduction of the exploration of the novel object in comparison with the ancient object (**Figure 3**). The discrimination index which is 0.62 ± 0.03 in distilled water group was significantly reduced (*P* < 0.0001) to a value of 0.014 ± 0.04 in the scopolamine-alone-treated group.

Ten (10) days pretreatment of mice with the decoction of *D. integrifolia* at the dose of 87.5 mg/kg significantly (*P* < 0.01) increased the exploration of the novel object in comparison with the ancient object. Thus, the discrimination index rose from a value of 0.014 ± 0.04 in the scopolamine-alone group to 0.46 ± 0.10 at the dose 87.5 mg/kg of *D. integrifolia*. During the retention phase, tacrine significantly (*p* < 0.01) increased the exploration of the novel object in comparison with the ancient object presented during the acquisition phase. The discrimination index of the tacrine group was 0.51 ± 0.80 (**Figure 3**).

### Effects of *D. integrifolia* on Acquisition and Retention Parameters of Scopolamine-Treated Mice in a Morris Water Maze Task

#### Acquisition

*Dichrocephala integrifolia* at all doses significantly reduced the acquisition deficit caused by scopolamine starting from the 2nd day of the acquisition phase (day 7) (*P* < 0.0001) (**Table 1**). The time to find the invisible platform in the 2nd day of the acquisition was 69.91 ± 4.41 s in the scopolamine alone against 14.58 ± 2.58 s at the dose of 87.5 of *D. integrifolia*.

**Table 1:** Effect of *Dichrocephala integrifolia* on Escape Latency (EL) of mice using morris water maze.

Treatments for 10 days	Dose (mg/kg)	EL (s) Day-6	EL (s) Day-7	EL (s) Day-8	EL (s) Day-9
DW + Sal	- + -	27.37 ± 4.09^∗^	13.62 ± 1.87^∗∗∗^	12.20 ± 2.58^∗∗∗^	9.75 ± 0.30^∗∗∗^
DW + Sco	- + 1	68.67 ± 12.03^a^	69.91 ± 4.14^c^	65.20 ± 3.38^c^	78.66 ± 3.75^c^
DI + Sco	35 + 1	70.96 ± 12.47^a^	39.54 ± 11.49^a∗∗^	29.50 ± 4.52^b^	29.04 ± 1.41^c∗∗∗^
DI + Sco	87.5 + 1	36.52 ± 8.78	14.58 ± 2.58^∗∗∗^	15.25 ± 0.67^∗∗∗^	13.25 ± 1.99^∗∗∗^
DI + Sco	175 + 1	41.58 ± 6.41	28.33 ± 0.98^∗∗∗^	24.66 ± 2.73^∗∗∗^	21.5 ± 2.01^b∗∗∗^
DI + Sco	350 + 1	36.54 ± 8.87	32.04 ± 2.88^∗∗∗^	14.67 ± 4.37^a∗∗∗^	19.91 ± 1.70^a∗∗∗^
Tac + Sco	10 + 1	26.29 ± 5.70^∗^	12.62 ± 2.46^∗∗∗^	10.87 ± 1.74^∗∗∗^	8.57 ± 1.88^∗∗∗^


*Results are expressed as mean ± SEM for six mice, ^∗^*p* < 0.05, ^∗∗^*p* < 0.01, ^∗∗∗^*p* < 0.001 compare to scopolamine alone treated group (DW + Sco). ^a^*p* < 0.05, ^b^*p* < 0.01, ^c^*p* < 0.0001 compare to control group (DW + Sal). *F*(6,35) = 4.366; *P* = 0.0022 (Day 6); *F*(6,35) = 16.67; *P* < 0.0001 (Day 7); *F*(6,35) = 16.67; *P* < 0.0001 (Day 8); *F*(6,35) = 136.26; *P* < 0.0001 (Day 9). DW, distilled water; DI, *D. integrifolia*; Sal, saline; Sco, scopolamine; Tac, tacrine.*

#### Retention

**FIGURE 4:**
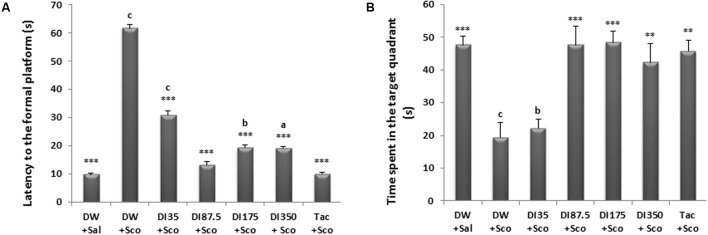
Effect of *D. integrifolia* on scopolamine-induced memory impairment on retention parameters in the morris water maze. Latency to the non-existing platform **(A)**. Time spent in the target quadrant **(B)**. Each column represents mean ± SEM of six animals. The data analysis was performed using one way ANOVA followed by Tukey multiple comparisons test ^∗∗^*P* < 0.01, ^∗∗∗^*P* < 0.001; vs. scopolamine-alone treated group (DW + Sco). ^a^*p* < 0.05, ^b^*p* < 0.01, ^c^*p* < 0.001 vs. distilled water group. DW, distilled water; DI, *D. integrifolia*; Sal, saline; Sco, scopolamine; Tac, tacrine.

As shown in **Figure 4A**, *D. integrifolia* at all doses and also tacrine 10 mg/kg significantly reduced the latency time to the non-existing platform on the retention phase when compared to scopolamine-alone treated group (*P* < 0.0001). The latency time to reach the non-existing platform was 61.66 ± 9.83 s in the scopolamine alone treated group against 13.16 ± 2.48 s at the dose 87.5 mg/kg of *D. integrifolia.* Furthermore, *D. integrifolia* from the dose of 87.5 mg/kg and tacrine significantly increased the time spent in the target quadrant during the retention phase when compared to scopolamine–alone treated group (*P* < 0.0001) (**Figure 4B**).

### Effects of *D. integrifolia* on Brain Acetylcholinesterase Activity, Malondialdehyde and Reduced Glutathione Levels

The activity on AChE was significantly reduced by *D. integrifolia* at the dose of 87.5 mg/kg, Tacrine also inhibited the effect of scopolamine induced increased in the activity of AChE when compared to scopolamine-alone treated group (*P* < 0.0001) (**Table 2**).

**Table 2:** Effects of *Dichrocephala integrifolia* on acetylcholinesterase activity, malondialdehyde and reduced glutathione levels of scopolamine treated mice.

Treatments (for 10 days)	Dose (mg/kg)	AchE (μmol/min/mg protein)	MDA (μmol/mg protein)	GSH (μmol/mg protein)
DW + Sal	– + –	11.98 ± 1.13^∗∗∗^	0.12 ± 0.04^∗∗∗^	20.00 ± 1.77^∗∗∗^
DW + Sco	– + 1	29.41 ± 6.42^c^	0.61 ± 0.12^c^	12.33 ± 1.05^c^
DI + Sco	35 + 1	22.89 ± 3.88^a^	0.53 ± 0.09^c^	13.00 ± 1.05^c^
DI + Sco	87.5 + 1	13.25 ± 3.88^∗∗∗^	0.29 ± 0.22^b∗∗^	24.66 ± 2.43^∗∗∗^
DI + Sco	175 + 1	19.19 ± 4.00^∗^	0.35 ± 0.09^∗^	20.33 ± 1.40^∗a^
DI + Sco	350 + 1	19.88 ± 6.64^∗^	0.28 ± 0.08^∗∗^	19.83 ± 1.81^a∗^
Tac + Sco	10 + 1	12.52 ± 7.46^∗∗∗^	0.21 ± 0.06^∗∗∗^	27.5 ± 0.67^∗∗∗^


*Results are expressed as mean ± SEM for six mice, ^∗^*p* < 0.05, ^∗∗^*p* < 0.001, ^∗∗∗^*p* < 0.0001 compare to scopolamine alone treated group (DW + Sco). ^a^*p* < 0.05, ^b^*p* < 0.001, ^c^*p* < 0.0001 compare to control mice (DW + Sal). DW, distilled water; DI, *D. integrifolia*; Sal, saline; Sco, scopolamine; Tac, tacrine.*

The co-administration of *D. integrifolia* from the dose 87.5 mg/kg and scopolamine significantly reduced the level of brain malondialdehyde when compared to scopolamine-alone treated group (*P* < 0.0001) (**Table 2**). Tacrine also reduced the level of MDA (**Table 2**).

The level of GSH was lower in the scopolamine -alone -treated group. The administration of the decoction of *D. integrifolia* from the dose of 87.5 mg/kg significantly (*P* < 0.0001) reverses the reduction of GSH induced by scopolamine (**Table 2**).

### Results of the Histopathological Studies

**FIGURE 5:**
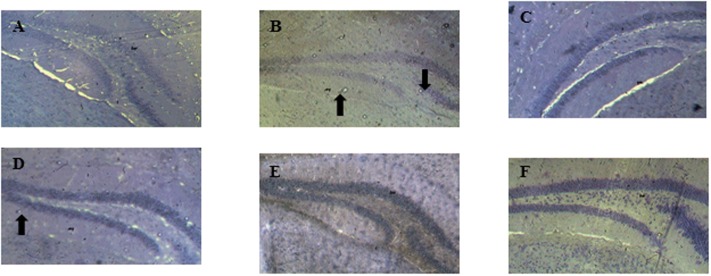
Effects of *D. integrifolia* on neuronal loss in the hippocampus dentate gyrus induced by scopolamine. Distilled water group **(A)**; scopolamine alone treated group **(B)**; *D. integrifolia* 87.5 mg/kg **(C)**; *D. integrifolia* 175 mg/kg **(D)**; *D. integrifolia* 350 mg/kg **(E)**; tacrine **(F)**. Nissl stain magnification (40).

The histopathological analysis show that the dentate gyrus of distilled water group of mice is normal without any sign of neurodegeneration or necrosis (**Figure 5A**). The hippocampal sections of scopolamine-treated group show a significant reduction in the density of cells of all the layers of the dentate gyrus associated with the presence of apoptotic cells (**Figure 5B**). The decoction of *D. integrifolia* at the doses of 87.5, 175, and 350 mg/kg show a normal architecture of the cells layer of the dentate gyrus (**Figures 5C–E**, respectively). Tacrine group shows a dentate gyrus without any sign of necrotic or apoptotic cells (**Figure 5F**).

## Discussion

Alzheimer’s disease is a deadly progressive neurodegenerative disorder of the elderly associated with loss of memory and cognitive dysfunctions (Behl, 2002; Madeo and Elsayad, 2013). Cumulative evidences have suggested that the cognitive symptoms of AD are a result of the impairment of adult neurogenesis in the hippocampus (Demars et al., 2010; Lazarov and Marr, 2013). AD has become a public health burden due to the increase of aging population and increase lifetime expectancy (Kalaria et al., 2008; Olayinka and Mbuyi, 2014).

In this study, we evaluated the effect of the decoction of *D. integrifolia* against scopolamine model of AD. Scopolamine is an alkaloid extracted from the Solanaceae *Datura stramonium* and which impairs short-term and long term-memory in animals and humans (Rabiei et al., 2015). Through the interference with acetylcholine in the brain, scopolamine can cause oxidative stress leading to cognitive impairment (Rahnama et al., 2015). Thus, scopolamine-induced memory impairment is a valid model for the evaluation of anti – amnesic effects of new drugs. Diverse behavioral animal models are usually used for the evaluation and validation of new drugs against dementia (Rajendran et al., 2014).

In the present study, 10 days pretreatment of animals with *D. integrifolia* significantly counteracted the reduction of the percentage of spontaneous alternation induced by scopolamine suggesting significant improvement of space related short – term memory by the decoction of *D. integrifolia* (Hritcu et al., 2012, 2015; Beppe et al., 2014). The effect of *D. integrifolia* and that of tacrine a cholinesterase inhibitor used in the treatment of AD were comparables.

The EPM test was used for the evaluation of learning and memory. The EPM is based on the apparent natural aversion of rodents to open and high spaces, and originally, it is used for measurement of anxiety (Pellow et al., 1985; Pellow and File, 1986; Lister, 1987). Some parameters of the EPM such as retention transfer latency (the time take by the animal to move from the open arms to the enclosed arms) is used for the evaluation of memory. And an animal that has previously (acquisition trial) experienced entering the open arms have the shortened transfer latency in the retention trial (Itoh et al., 1990). In the EPM test, *D. integrifolia* significantly reduced the initial transfer latency on day 10. These results suggest that *D. integrifolia* has a nootropic effect because it ameliorates the retention of informations in absence of any memory impairment inducer (Itoh et al., 1990; Rajendran et al., 2014). Furthermore, *D. integrifolia* significantly reduced the retention transfer latency on day 10 after scopolamine injection suggesting that *D. integrifolia* ameliorates learning and retention of information and plays a role in memory formation (Itoh et al., 1990; Sharma and Kulkarni, 1992; Joshi and Parle, 2007; Kulkarni et al., 2011; Rajendran et al., 2014).

The NOR test was used to evaluate the effect of *D. integrifolia* on recognition memory. We found that, 10 days pretreatment of mice with *D. integrifolia* significantly reversed the reduction of the discrimination index induced by scopolamine suggesting the effect of the plant on recognition memory (Ennaceur and Delacour, 1988). All these above results clearly show that *D. integrifolia* has a neuroprotective activity *in vivo* by counteracting memory impairment induced by scopolamine in a variety of behavioral paradigms.

To delineate the mechanism by which *D. integrifolia* exerts his neuroprotective activity, *D. integrifolia* was administered during 10 days consecutively 1 h prior scopolamine (1 mg/kg i.p.) injection. The MWM task was used as behavioral task. The results obtained show that like tacrine, *D. integrifolia* significantly reduced the learning and retention deficits caused by repeated does of scopolamine. *D. integrifolia* reduced the time to the invisible platform during acquisition and the latency time to the non-existing platform during retention phase. *D. integrifolia* also significantly increased the time spent in the target quadrant during this retention phase. Our results with the MWM suggest that *D. integrifolia* improves spatial long-term memory (Cheng et al., 2011). The results of MWM confirmed that pretreatment with *D. integrifolia* counteracted scopolamine induced learning and memory deficit thus *D. integrifolia* is neuroprotective (Konar et al., 2011; Hritcu et al., 2015).

The results of biochemical assays show that 10 days administration of scopolamine increased the activity of AChE and the level of MDA, a measure of brain lipid peroxidation (Hritcu et al., 2015) and reduced the level of GSH, the main antioxidant enzyme of the body (Agarwal et al., 2011). Our results are in accordance with literature that shows that administration of scopolamine in rodents can lead to increase of AChE activity and oxidative status in the brain (Cheng et al., 2011; Konar et al., 2011; Baradaran et al., 2012; Rajendran et al., 2014). Pretreatment of mice with *D. integrifolia* reversed the increase of the activity of AChE and oxidative stress induced by scopolamine, thus protecting animals against learning and memory loss.

The results of histopathological studies demonstrated that 10 days administration of scopolamine resulted in neurodegenerative processes in the dentate gyrus when compared to naïve mice.

This cell death in the hippocampus dentate gyrus was significantly prevented by a pretreatment with *D. integrifolia*. The dentate gyrus is the part of the brain where adult neurogenesis takes place and it is also implicated in hippocampal neurogenesis and plasticity (Kempermann et al., 2015). Besides the facts that memory impairment induced by scopolamine is a result of an increase in AChE activity and brain oxidative status (Chen et al., 2008; Konar et al., 2011), it can be also assumed that scopolamine impairs neurogenesis in the brain which in turn leads to cognitive deficits as in AD (Demars et al., 2010; Lazarov and Marr, 2013). By antagonizing the cell death in the dentate gyrus induced by scopolamine, *D. integrifolia* can be a good treatment for cognitive deficits and AD. There are cumulative evidences in literature that scopolamine influences acquisition, consolidation and recall of informations and that scopolamine is a cholinergic blocker (Agrawal et al., 2009; Konar et al., 2011; Rajendran et al., 2014). By counteracting the effect of scopolamine, *D. integrifolia* can have the same mechanism of action, as tacrine wish is a cholinergic enhancer widely used in the treatment of AD. Furthermore, *D. integrifolia* had demonstrated many beneficial effects against others diseases such as hepatotoxicity probably due to its antioxidant properties (Ngueguim et al., 2016). This property of the plant may also be strongly involved in its neuroprotective effects observed in our study. This study shows that *D. integrifolia* has an ability to improve learning of information, ameliorates spatial short-term and long-term memory and recognition memory. The mechanism by which *D. integrifolia* exerts its effects may be related to the reduction of AChE level associated with antioxidant properties and improvement of adult neurogenesis. The overall results of this study can explain the wide usage of *D. integrifolia* in the treatment of dementia in Central Africa.

## Conclusion

The results of this study shows that the decoction of *D. integrifolia* counteracted scopolamine-induced memory impairment and oxidative stress. Thus, it can be concluded that *D. integrifolia* can be a valuable plant resource for the management of dementia in general an age-related cognitive deficit of Alzheimer’s type in particular. Nevertheless, more studies with *D. integrifolia* targeting other hypotheses of AD are needed to clarify the exact mechanism of action of the plant.

## Author Contributions

NK, GT, SP, FM, GTN, and EB conceived and designed the work. NK, GT, SP, JN, GCNN, and DP collected and analyzed the data. NK, GT, and EB wrote and revised the manuscript. All authors read and approved the final manuscript.

## Conflict of Interest Statement

The authors declare that the research was conducted in the absence of any commercial or financial relationships that could be construed as a potential conflict of interest.
